# Advances in pulmonary neuroendocrine cells and their cellular interactions in asthma pathogenesis

**DOI:** 10.3389/fphar.2025.1725531

**Published:** 2026-01-07

**Authors:** Lanlan Wang, Hui Wang, Yamei Yuan, Weidong Ye, Xiangming Fang

**Affiliations:** Anhui University of Chinese Medicine, Hefei, China

**Keywords:** asthma, eosinophils, goblet cell, ILC2s, PNECs

## Abstract

Asthma is a complex chronic inflammatory airway disease, whose pathogenesis involves interactions among multiple cell types and molecular pathways. Pulmonary neuroendocrine cells (PNECs), rare epithelial cells in the lungs, have recently gained attention as pulmonary sensors. PNECs detect environmental signals and regulate pulmonary immune responses and physiological functions by releasing neuropeptides and neurotransmitters. Aberrant activation of PNECs is implicated in various respiratory diseases, including asthma, where they interact significantly with other cell types, such as type 2 innate lymphoid cells (ILC2s), goblet cells, and eosinophils. Recent studies have highlighted the potential of targeting PNEC-related pathways for developing novel anti-inflammatory drugs and identifying biomarkers to evaluate drug efficacy and toxicity in asthma. Exploring asthma pathogenesis through the functional characteristics of PNECs and their interactions with other cells could have potential clinical value in diagnosing and treating of asthma.

## 1 Introduction

Asthma is a common chronic respiratory disease with prevalence rates that vary significantly across different countries and regions. Despite considerable advances in asthma management, the prevalence, incidence, and mortality rates continue to rise, imposing a substantial burden on individuals and public health systems (Zhang L. et al., 2025).

Asthma is characterized by inflammatory responses, airway remodeling, and airway hyperresponsiveness (Song et al., 2025). Airway epithelial and smooth muscle cells are pivotal to its pathogenesis. Airway epithelial cells serve as the primary barrier against inhaled pathogens and particles, triggering inflammation and mucus production, both of which contribute to airway obstruction. Another significant cause of airway obstruction is bronchoconstriction mediated by airway smooth muscle cells (Raby et al., 2023). Asthma does not present as a single symptom but rather encompasses multiple syndromes with distinct etiologies, pathophysiological mechanisms, clinical manifestations, and therapeutic responses. Its causes are diverse: allergic asthma is triggered by allergens (e.g., house dust mites, pollen, pet dander) and is typically associated with elevated specific IgE levels; non-allergic asthma is unrelated to allergies and can be triggered by infection, air pollution, or occupational exposures. Other forms include exercise-induced asthma, drug-induced asthma and obesity-related asthma (Imayama et al., 2024). The pathophysiology of asthma is multifaceted. Type 2 inflammatory asthma is characterized by Th2 cells, ILC2s, eosinophils and IgE, typically linked to allergic asthma. Non-type 2 inflammatory asthma involves neutrophils, Th1/Th17 cells, or paucigranulocytic patterns, frequently associated with more severe disease and corticosteroid resistance (Matucci et al., 2021; Fuhlbrigge and Sharma, 2025). Clinical manifestations vary in severity, ranging from mild intermittent episodes to severe persistent symptoms. Attack frequency also varies considerably, from occasional to frequent occurrences. Lung function impairment differs in severity, potentially resulting in significant airflow limitation in severe cases. Asthma management emphasises individualised treatment. For instance, some patients respond well to inhaled corticosteroids (ICS), whereas others exhibit steroid resistance. Responses to specific biologics (e.g., anti-IgE, anti-IL-5 therapies) also vary markedly among asthma subtypes (D’Amato et al., 2025; Pelaia et al., 2020). Consequently, asthma research necessitates greater precision.

**FIGURE 1:**
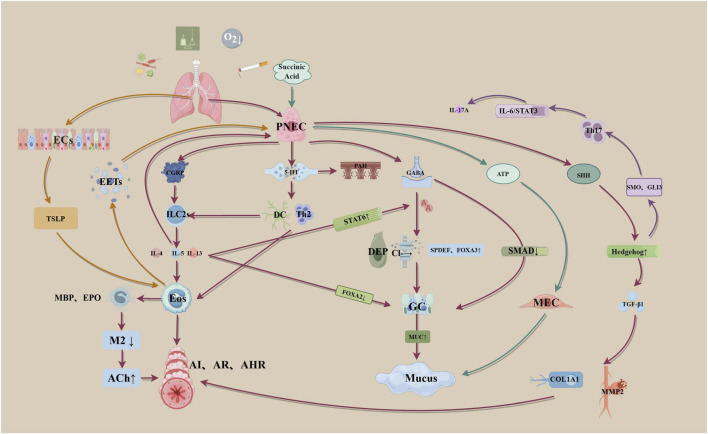
Conceptual Framework for Multi-cellular Interactions of PNEC-derived Mediators in Asthma (By Figdraw). Abbreviations: PNEC, Pulmonary neuroendocrine cell; CGRP, Calcitonin gene-related peptide: ILC2s,type 2 innate lymphoid cells; IL, Interleukin; Eos, Eosinophils; Al, Airway Inflammation; AR, Airway Remodelling; AHR, Airway Hyper-responsiveness; MBP, Major basic protein; EPO, Eosinophil Peroxidase; M2. Muscarinic Acetylcholine Receptor Type 2; ACh, Acetylcholine; 5-HT, Serotonin: DC, Dendritic Cell; Th2, T helper type 2 cell; PAH, Pulmonary Arterial Hypertension: GABA, Gamma-aminobutyric acid; STAT, Signal Transducer and Activator of Transcription; FOXA, Forkhead Box A; SPDEF,SAM Pointed Domain-containing ETS Transcription Factor; DEP, Depolarization: GC, Goblet cell; MUC, Mucin; SHH, Sonic Hedgehog; TGF-β1, Transforming Growth Factor Beta 1; COL1A1, Collagen Type I Alpha 1 Chain; MMP2, Matrix Metalloproteinase 2; ECs, Epithelial Cells; TSLP, Thymic stromal lymphopoietin; EETS, Eosinophil extracellular traps:SMO, Smoothened; GLI3, Glioma-Associated Oncogene Homolog 3: ATP, Adenosine Triphosphate; MEC, Myoepithelial Cell.

Although PNECs constitute only approximately 0.5% of the lung epithelium, they play a crucial role in respiratory health and disease (Candeli and Dayton, 2024). As airway sensors, PNECs detect environmental stimuli and modulate immune cell activity by releasing neuropeptides such as calcitonin gene-related peptide (CGRP) and gamma-aminobutyric acid (GABA), This triggers pulmonary immune responses, influencing the symptoms and progression of various lung diseases. Particularly in allergic asthma, PNECs amplify immune reactions by stimulating ILC2s (Sui et al., 2018; (Branchfield et al., 2016). Furthermore, PNECs are critical in lung development and regeneration, with the ability to transdifferentiate into other cell types, such as Clara cells and ciliated cells, following lung injury (Candeli and Dayton, 2024). The role of PNECs in asthma involves complex intercellular interactions. Cells such as ILC2s, goblet cells, and eosinophils interact with PNECs to varying degrees, collectively influencing asthma pathogenesis and progression. Therefore, trageting PNECs for asthma treatment offers novel therapeutic avenues (Figure 1).

## 2 Overview of PNECs

PNECs exhibit distinctive distribution and morphology, with primary roles in chemosensation, immunoregulation, regeneration and repair, and neuromodulation. Crucial signalling pathways associated with PNECs include the Notch and Hedgehog pathways.

### 2.1 Distribution and morphology of PNECs

PNECs constitute a distinct epithelial cell type lining the airways of varying sizes, maintaining contact with sensory nerve fibers (Seeholzer and Julius, 2024). Within the airway epithelium, PNECs exist as solitary cells or small clusters, interspersed between the two primary airway epithelial cell types:secretory Clara cells and ciliated cells (Rawlins et al., 2009). Neuroepithelial bodies (NEBs) are cellular clusters consisting of PNEC aggregates (20–30 cells), bearing membrane receptors sensitive to numerous stimuli, including hypoxia and nicotine. Isolated PNECs typically exhibit a flask-shaped or elongated morphology. Under scanning electron microscopy, NEBs form crater-like depressions lined with microvilli, surrounded by non-ciliated cells with smooth surfaces at their periphery. Stereo-photmicroographs reveal that these surrounding cells project above the adjacent bronchioles mucosa (Noguchi et al., 2020; De Proost et al., 2008). NEBs have direct contact with the airway lumen and are exclusively located at airway bifurcations, whereas isolated PNECs or smaller clusters are scattered throughout the bronchial epithelium. This distribution pattern highlights their crucial role in pulmonary function and disease (Gilmour, 2015). and reflects their unique function as intrapulmonary sensors.

### 2.2 Primary functions of PNECs

#### 2.2.1 Chemosensory function

PNECs rapidly detect changes in oxygen levels and respond to hypoxic environments, a capability seemingly linked to their distinct localization at airway bifurcations. During hypoxia, they express and secrete serotonin (5-hydroxytryptamine; 5-HT) (Fu et al., 2002), a monoamine neurotransmitter that induces pulmonary vasoconstriction. The localised hypoventilation observed in bronchoconstrictive asthma patients spatially correlates with reduced perfusion, a phenomenon attributed to hypoxic pulmonary vasoconstriction (HPV) (Kelly et al., 2017). Dense cisternae vesicles (DCVs) are present in the cytoplasm of NEB cells, storing neurotransmitters, amines, peptides, and other substances. Hypoxic conditions can trigger DCV degranulation, releasing these substances in response to environmental changes. Furthermore, NEBs express several essential proteins that collectively constitute the functional NADPH oxidase complex. under hypoxic conditions, K+ channels on PNECs close, whereas voltage-gated Ca2+ channel open, facilitating extracellular Ca2+influx and inducing Ca2+-dependent exocytosis of DCVs (Youngson et al., 1993). This suggests that NEBs may serve as hypoxia-sensitive airway chemical sensors in respiratory regulation.

PNECs can detect nicotine, which induces acute asthma attacks and promotes pulmonary edema, lung injury, and abnormal leukocytosi, thereby exerting detrimental effects on both pulmonary and systemic functions (Ahmad et al., 2019). Nicotine,an agonist of nicotinic acetylcholine receptors (nAChRs), and its nitrosamine derivative NNK can induce PNEC hyperplasia in asthma by upregulating α7-nicotinic acetylcholine receptors (α7-nAChR) in PNECs. These proliferated PNECs subsequently induce pulmonary hypertension through secretion of 5-HT (Schuller et al., 2003).

Beyond sensing hypoxia and nicotine stimulation, PNECs also respond to mechanical changes. Mechanical stretching stimulates PNECs to release 5-HT, thereby influencing lung development (Pan et al., 2006).

#### 2.2.2 Immunomodulation

PNECs are neuroendocrine cells possessing dual neurosensory and endocrine functions (Xu et al., 2020). Using genetically engineered knockout mice, Branchfield et al. demonstrated that inactivation of the Roundabout gene in PNECs disrupts normal aggregation of and enhances neuropeptide production, triggering an elevated immune response. The sequence of events—elevated neuropeptide secretion from PNECs, increased immune cell infiltration, and lung architectural remodeling—indicates that PNECs, despite their scarcity, function as sensitive and effective variable resistors within the airway wall. They receive, interpret, and respond to environmental stimuli, initiating immune responses via neuropeptides, and thereby exert immunomodulatory effects (Branchfield et al., 2016).

Achaete-scute-like protein 1 (Ascl1), a basic helix-loop-helix transcription factor, serves as an early determinant of PNEC fate. Sui et al. generated mouse mutants lacking PNECs by inactivating Ascl1 in airway epithelium. Following allergen challenge, reduced secretion of PNEC-derived substances (such as CGRP and GABA), severe diminishment of goblet cell proliferation, decreased immune cell infiltration, and suppressed type 2 (allergic) immune responses were observed in Ascl1-mutant mice. Additionally, ILC2s, like PNECs, accumulate at airway branching points. Subsequent investigations revealed that stimulating mouse ILC2s with CGRP induces interleukin-5 (IL-5) production, whereas inactivation of the CGRP receptor gene Calcrl in ILC2s was attenuates their response to allergens. This indicates that CGRP, a product of PNECs, induces cytokine secretion in ILC2s. Thus, PNECs enhance ILC2s activity by secreting CGRP, thereby recruiting downstream immune cells. Whilst GABA does not induce cytokine secretion in ILC2s as CGRP does,allergen challenge in GABA-inactive knockout mice (Gad1CKO mice) showed reduced goblet cell proliferation. This indicates that GABA can promote goblet cell proliferation and increase mucus secretion. Further research demonstrated that instilling a mixture of CGRP and GABA in mouse mutants with specifically ablated PNECs (Ascl1CKO mice) restores both the immune cell infiltration and goblet cell proliferation. This demonstrates that CGRP and GABA are the primary molecular effectors through which PNECs exert their immune functions *in vivo* (Sui et al., 2018).

It should be noted that the current conclusions are primarily based on mouse experiments, and direct human evidence remains lacking. Although mouse models are powerful tools for studying asthma mechanisms, differences in PNEC distribution, abundance, and immune microenvironment between humans and mice may exist.

#### 2.2.3 Regeneration and repair

PNECs play a pivotal role in lung regeneration and repair following injury. They not only self-renew but also differentiate into other cell types to support epithelial restoration. The stem cell function of PNECs is regulated by multiple genes, including the tumor suppressors Rb and P53, which inhibit excessive self-renewal. During airway injury, the Notch pathway is activated, initiating cellular deprogramming and migration to form large regenerative epithelial clonal patches that are essential for pulmonary environmental responses. It should be noted that not all PNECs exhibit stem cell activity; typically, only 2–4 cells per NEB possess reserve stem cell potential (Ouadah et al., 2019).

NEBs are considered a reserve niche for airway epithelial regeneration and contribute to local repair following Clara cell depletion. Reynolds et al., using an animal model of airway injury and repair, observed that epithelial regeneration after naphthalene-induced Clara cell ablation of preferentially occurred at airway branching points, resulting in the formation of new Clara cells. Expression of Clara cell secretory protein (CCSP) facilitated airway repair and NEB-associated proliferation, indicating that sustained regeneration is accompanied by NEB expansion. This suggests that the NEB microenvironment serves as an airway progenitor reservoir supporting focal epithelial regeneration (Reynolds et al., 2000a). NEBs constitute a highly dynamic structure that responds to chronic airway injury through PNEC proliferation. However, PNECs themselves function as a self-renewing progenitor population, and NEBs-associated Clara cells are not required for proliferative responses to chronic injury (Reynolds et al., 2000b).

#### 2.2.4 Neuromodulation

PNECs lie in close proximity to neural and immune cells, forming neuroimmune modules that enable the lung to sense and respond to environmental cues. PNECs are innervated (Kuo and Krasnow, 2015; (Brouns et al., 2009) and may participate in neurosecretory processes within the mucosal bronchioles and bronchioles (Popko et al., 2020) forming synaptic contacts with afferent and efferent nerve fibers. NEBs can transmit neural impulses to the central nervous system, functioning as neuroreceptors that mediate axon reflex-driven local regulation (Cutz et al., 2013). Moninger et al. proposed that PNECs possess latent sensory capabilities linked to the vagus nerve and immune system. Submucosal glands (SMGs), which produce protective mucus, contain PNECs that detect succinate, an inflammatory metabolite in airway surface fluid. In response, release ATP to stimulate myoepithelial cell contraction, thereby promoting mucus expulsion onto the airway surface. While essential for host defence, excessive mucus secretion may aggravate pulmonary disease. Thus, PNEC-mediated activation of SMGs in response to inflammatory signals significantly contributes to respiratory host defense (Yu et al., 2022)^,^ (Chang et al., 2015), forming part of the immunomodulatory response to inflammation and infection.

PNECs are the only pulmonary epithelial cells with direct neuronal innervation and play an crucial role in airway-nerve communication through neural pathways and hormone release, with potential implications for neuroprotection in diseases such as asthma. By integrating neuronal and endocrine functions, PNECs secrete neuropeptides upon stimulation that travel via the vagus nerve to the brain, where they modulate brainstem respiratory centers (Branchfield et al., 2016). In exercise-induced asthma, PNECs can detect changes in airways osmotic pressure and temperature, triggering reflex parasympathetic activation and vagus nerve-mediated bronchoconstriction (Thakur et al., 2024).

### 2.3 Primary signalling pathways involving PNECs

#### 2.3.1 Notch signalling pathway

The differentiation of PNECs is regulated by basic helix-loop-helix (bHLH) transcription factors such as Mash1 and Hes1. The Notch signaling pathway exerts a pivotal role in PNECs differentiation through regulation of Hes1 expression. Studies indicate that mice deficient in Mash1 exhibit an absence of PNECs, while those deficient in Hes1 display excessive PNEC proliferation. Thus, Mash1 serves as a marker for PNECs, whereas Hes1 is expressed primarily in non-neuroendocrine cells (Ito et al., 2003). Furthermore, immunohistochemical analysis detects Notch1, Notch2, and Notch3 in non-neuroendocrine cells, with Notch1 mRNA expression being dependent on Hes1 (Ito et al., 2000). These findings suggest that the balance between PNECs and non-neuroendocrine cell formation is tightly regulated by Notch signaling.

#### 2.3.2 Hedgehog signalling pathway

The Hedgehog signaling pathway contributes to PNEC of differentiation and the development of small cell lung cancer (SCLC). Activation of this pathway occurs during lung injury repair and normal differentiation of neuroendocrine precursor cells (Watkins et al., 2003), promoting epithelial-to-goblet cell transformation (Xu et al., 2018). ICS primarily suppress lymphocyte and eosinophil activity but exert limited effects on upstream epithelial signalling pathways, such as Hedgehog signalling, and the neurosecretory functions of PNECs. This partially explains the ineffectiveness of ICS in preventing airway remodelling in certain patients. Consequently, targeting the Hedgehog pathway represents a promising strategy for early intervention in chronic airway diseases.

The Hedgehog signalling pathway is activated in the airways of children with allergic asthma. In eosinophilic asthma, PNECs activate Hedgehog signalling through secretion of ligands such as Sonic Hedgehog (SHH), promoting TGF-β1-mediated collagen (COL1A1) and MMP2 expression. Increased COL1A1 expression, partially mediated by the PTCH1–SMO–GLI axis of the Hedgehog pathway, contributes to airway fibrosis and remodelling. Thus, Hedgehog signalling represents a potential therapeutic target for chronic airway diseases, including asthma. GLI inhibitors (e.g., GANT61) suppress downstream fibrotic and inflammatory gene expression by targeting GLI1/2 transcription factors, reducing excessive extracellular matrix synthesis and deposition (Tam et al., 2022).

In neutrophilic asthma, the Hedgehog pathway facilitates Th17 cell differentiation via Smo and GLI3, dependent on the IL-6/STAT3 signalling axis, thereby upregulating inflammatory mediators such as IL-17A. This asthma phenotype often demonstrates glucocorticoid resistance. Experimental studies have shown that Smo and GLI3 are upregulated during ***in vitro*** Th17 polarisation and *in vivo* models of Th17-dominant asthma. Inhibition of Smo with small-molecule inhibitors or knockdown of GLI3 suppresses Th17 polarisation. Furthermore, inhibiting Smo affects the polarisation of not only Th17 cells but also Th1, Th2, and Treg cells. GLI3 directly interacts with IL-6 in T cells, inducing STAT3 phosphorylation and promoting Th17 cell differentiation; indeed, IL-6 is essential for GLI3-mediated regulation of Th17 polarisation. Additionally, clinical data have confirmed correlations between elevated GLI3 expression and increased levels of IL-17A and IL-6 in asthmatic children. These findings provide molecular insights into the interaction between Hedgehog signalling and Th17 differentiation, highlighting Smo and GLI3 as potential therapeutic targets for corticosteroid-resistant asthma driven by Th17 cells (Jin et al., 2024). Smo inhibitors (such as Vismodegib and Criptobanil) can reduce airway inflammation and remodelling by targeting the Hedgehog pathway, thereby alleviating various asthma phenotypes (Th2/Th17-type) (El-Baz et al., 2022).

## 3 Interaction between PNECs and immune cells

Direct or indirect interactions occur between PNECs and various immune cells, including ILC2s, goblet cells, and eosinophils. These cells, together with their interactions, play crucial roles in the pathogenesis and progression of eosinophilic asthma.

### 3.1 PNECs and ILC2s

#### 3.1.1 ILC2s and asthma

ILC2s represent a key class of immune cells predominantly located in mucosal tissues. Capable of rapid responses to allergens and environmental stimuli, they play multiple roles in asthma pathogenesis. They induce airway inflammation by producing large quantities of type 2 cytokines, such as IL-5 and IL-13, thereby triggering eosinophilic inflammation and airway hyperresponsiveness. These cytokines promote airway remodeling and mucus secretion, ultimately leading to airway obstruction and exacerbated symptoms (Kabata et al., 2015). Furthermore, ILC2s compromise bronchial epithelial barrier integrity through IL-13 (Sugita et al., 2018). They also synergise with Th2 cells via direct and indirect interactions to orchestrate type 2 immune responses (Matsuyama et al., 2023), thus playing a pivotal role in the immunopathology of asthma.

#### 3.1.2 Interactions between PNECs and ILC2s

During asthma development, PNECs stimulate ILC2s to produce IL-5 either through direct cell–cell contact or via secretion of the neuropeptide CGRP. These cytokines subsequently recruit downstream immune cells, amplifying the inflammatory cascade. Concurrently, ILC2s feedback-regulate PNEC activity by releasing cytokines. PNECs and ILC2s form neuroimmune modules at airway branching points, where they detect inhaled allergens and amplify these signals into robust pulmonary allergic responses. Environmental triggers, such as allergens, viral infections, and air pollution, may worsen asthma symptoms by activating the PNECs-ILC2s axis. This axis contributes to asthma pathogenesis and progression by promoting type 2 inflammation, enhancing airway hyperresponsiveness, and facilitating airway remodeling (Sui et al., 2018).

### 3.2 PNECs and goblet cells

#### 3.2.1 Goblet cells and asthma

Goblet cells are a major epithelial cell type responsible for secreting mucus to protect and lubricate the airway surfaces. In asthma, goblet cell number and function undergo substantial alterations. Abnormal goblet cell proliferation, along with changes in mucin (MUC) storage and secretion, contributes to key pathological features, including excessive mucus production and airway obstruction. Goblet cell hyperplasia is a well-established hallmark of asthma (Fahy, 2002; Ordoñez et al., 2001). Cytokines such as IL-13 and IL-4 promote goblet cell transdifferentiation and proliferation through induction of Th2-type immune responses. These cytokines exacerbate airway narrowing and clinical symptoms by increasing mucus storage secretion through MUC gene upregulation (Sun et al., 2017; Sahnoon et al., 2025).

#### 3.2.2 Interactions between PNECs and goblet cells

Interactions between PNECs and goblet cells in asthma largely involve the modulation of neurotransmission and immune signalling. PNECs regulate airway epithelial and immune cell activity by secreting molecules such as GABA and CGRP, thereby promoting the pathological airway changes.

PNECs are the primary source of GABA in the lung. GABA acts on airway epithelial cells via GABA_A and GABA_B receptors. Binding of GABA to GABA_A receptors activates Cl^−^ channels, leading to cellular depolarisation and Cl^−^ efflux, which promotes epithelial proliferation and differentiation into goblet cells. In asthma, expression of GABA_A and GABA_B receptor subtypes is elevated compared to healthy individuals. In a model of mucus-overproducing human bronchial epithelial cells, pharmacological blockade of GABA_A and GABA_B signaling reversed IL-13-mediated MUC5AC expression and goblet cell proliferation (Barrios et al., 2019). Further inhibition of GABA signalling reduced airway epithelial swelling, goblet cell hyperplasia, mucus secretion, and IL-13 levels simultaneously (Xiang et al., 2007). PNECs also enhance ILC2 activity by secreting CGRP, which promotes IL-13 production, activates the STAT6 signalling pathway, and upregulates GABA receptor expression. This synergises with GABA signalling to induce transcription factors SPDEF and FOXA3. SPDEF is a core driver of goblet cell differentiation, directly inducing mucin gene expression (e.g., MUC5AC) and promoting the goblet cell phenotype. FOXA3 similarly facilitates goblet cell differentiation, whereas FOXA2 is inhibited by IL-13; relieving this inhibition enhances goblet cell maturation and mucin production (Kimura et al., 2023). Thus, PNECs promote goblet cell proliferation and excessive mucus secretion through coordinated GABA and CGRP signalling, synergising with IL-13. Moreover, increased PNEC proliferation activates the GABAergic system, which enhances goblet cell proliferation and hypertrophy by suppressing the SMAD signalling pathway, thereby removing constraints on goblet cell differentiation (Piao et al., 2021; Feldman et al., 2019).

### 3.3 PNECs and eosinophils

#### 3.3.1 Eosinophils and asthma

Eosinophils are granulocytes containing eosinophilic granules that play significant role in allergic diseases, especially allergic eosinophilic asthma. They participate in allergic inflammatory responses not only as important effector cells but also as antigen-presenting cells (Choi, 2021). The mechanism underlying eosinophil involvement in asthma include their activation and migration within the airways. Cytokines produced by Th2 cells (e.g., IL-5 and IL-13) and chemokines (e.g., eosinophil chemotactic factor) directly mediate eosinophil recruitment to the lungs (Possa et al., 2013).

Eosinophils may exacerbate bronchoconstriction through neuroimmune interactions, thereby influencing asthma pathogenesis (Mattes and Collison, 2020). Firstly, eosinophils increase airway sensory nerve density and enhance nerve sensitivity by lowering neuronal activation thresholds, promoting nerve growth, and altering neuropeptide expression, thus driving airway hyperresponsiveness and bronchoconstriction (Drake et al., 2018a). Secondly, M2 muscarinic receptors on parasympathetic nerves play a critical role in regulating acetylcholine release in airway cholinergic nerves (Ghosh et al., 2025). Normally, M2 receptors modulate airway contraction by inhibiting acetylcholine release; however, this feedback mechanism is impaired in asthma (Drake et al., 2018b). Eosinophils antagonize M2 receptors by releasing major basic proteins (MBPs) and peroxidase (EPO), which function as endogenous allosteric antagonists of M2 receptors, thereby impairing their inhibitory function (Costello et al., 1998)^,^ (Jacoby et al., 1993), This leads to increased acetylcholine release and bronchial hyperreactivity (Fryer et al., 1999). Eosinophils also significantly contribute to inflammatory responses and airway remodeling by releasing pro-inflammatory mediators, including cationic proteins, cytokines, and chemokines (McBrien and Menzies-Gow, 2017). Eosinophil programmed cell death, such as necroptosis, is closely linked to inflammation and airway remodeling in asthma (He et al., 2022).

#### 3.3.2 Interactions between PNECs and eosinophils

Interactions between PNECs and eosinophils amplify allergic asthma responses at airway branching points via neuroimmune modules, which sense inhaled allergens and convert these stimuli into pulmonary immune responses.

Eosinophil extracellular traps (EETs), composed of DNA fibers and cytotoxic granulin proteins, are implicated in asthma pathogenesis (Choi et al., 2018), with excessive EET production potentially contributing to chronic inflammation and autoimmunity (Mukherjee et al., 2018). Thymic stromal lymphopoietin (TSLP), secreted by epithelial cells in response to environmental stimuli, activates cells of the innate and adaptive immune systems. Eosinophils respond to TSLP stimulation by producing EETs (Morshed et al., 2012), which in turn activate PNECs. PNEC activation further amplifies immune responses through secretion of neuropeptides and neurotransmitters, possibly via the CCDC25-ILK-PKCα-CRTC1 pathway (Lu et al., 2021).

## 4 Pathological mechanisms of PNECs in asthma

Asthma can be classified into eosinophilic (allergic), neutrophilic, mixed-cell, and oligocellular phenotypes. The mechanisms by which PNECs and their secreted neuropeptides and neurotransmitters function differ across asthma phenotypes and exhibit phenotype specificity. Mediators such as 5-HT, CGRP, and GABA are particularly crucial in allergic (type 2) asthma pathogenesis. CGRP and GABA respectively promote immune cell activation and goblet cell hyperplasia, exacerbating airway inflammation and mucus secretion. Thus, targeting PNECs and their signalling molecules represents a promising strategy for precision asthma therapies (Table 1).

**TABLE 1:** Summary of key signaling molecules, pathways, and targeted therapeutic strategies in asthma pathophysiology.

Category	Molecule/Pathway	Mechanism of action	Overall effect in asthma	Targeted therapy and drug development	References
Neuropeptide	SP and NKA	Exerts pro-inflammatory effects in epithelial and immune cells via binding to NK-1R	Promote inflammation	NK-1R antagonists (e.g., aprepitant) are under investigation; however, their efficacy in asthma remains uncertain	(O'Connor et al., 2004), (Li et al., 2022)
​	NKA	Induces airway responses via NK-2R	Promotes AHR	NK-2R antagonists (e.g., SR 48968) are undergoing preclinical or early clinical evaluation to counteract bronchoconstriction induced by NKA.	Groneberg et al. (2004)
​	VIP	Mediates immune responses by interacting with specific immune cell receptors	Reduces inflammation	VIP and its analogues are considered potential anti-inflammatory agents, although their stability, mode of administration, and effectiveness require further optimisation	(Misaka et al., 2010), (Verma et al., 2017)
​	N/OFQ	Exhibits anti-inflammatory properties in severe asthma	Reduces inflammation	N/OFQ receptor agonists represent novel anti-inflammatory agents, primarily explored in preclinical studies	D’Agostino et al. (2019)
​	CGRP	Through the CLR/RAMP1 receptor, CGRP either promotes inflammation by enhancing ILC2 activity or inhibits inflammation as a negative regulator of ILC2, depending significantly on the tissue microenvironment	Bidirectional regulation of inflammation	CGRP receptor antagonists (e.g., rimegepant) suppress ILC2 responses in asthma models, but their potential for inflammatory complications necessitates careful evaluation	(Li et al., 2014), (Ike et al., 2023), (Zhang R.et al., 2025)
Neurotransmitter	5-HT	May promote inflammation via certain pathways or exert anti-inflammatory effects through specific receptors (e.g., 5-HT2A) Exposure upregulates TPH1 and TPH2, increasing 5-HT levels and triggering pulmonary arterial hypertension	Bidirectional regulation of inflammation, Promotes AHR	5-HT2A receptor antagonists and TPH inhibitors (e.g., LX1032) are being explored to inhibit **5-HT** synthesis or pathogenic signalling, thereby mitigating airway hyperresponsiveness	(Flanagan et al., 2017), (Nau et al., 2012), (Flanagan et al., 2024), (Lu et al., 2025)
​	GABA	Effects vary based on receptor subtype, cellular context, and inflammatory milieu	Bidirectional regulation of inflammation	GABA_A receptor agonists (e.g., oral compound MIDD0301) demonstrated promising outcomes in preclinical studies, reducing airway inflammation and hyperresponsiveness with minimal adverse effects	(Gong et al., 2023), (Zahn et al., 2019), (Yocum et al., 2017), (Shirey et al., 2023)
Signalling pathway	Notch signalling	Downregulation promotes PNEC proliferation	Inhibit airway remodelling, alleviates AHR	Notch signalling agonists are potential therapeutic candidates for reducing airway remodelling by inhibiting PNEC proliferation, but most remain in the preclinical stage	Kim et al. (2025)
​	Hedgehog signalling	Eosinophil-like phenotype: PNECs secrete SHH, enhancing TGF-β1-induced COL1A1 and MMP2 via the PTCH1-SMO-GLI axis, driving fibrosis Neutrophil-like phenotype: Promotes Th17 differentiation via IL-6/STAT3 signalling involving Smo and GLI3, increasing IL-17A and causing hormone insensitivity	Promotes airway remodelling and inflammation	SMO inhibitors (e.g., Vismodegib and Cyclopamine) and GLI inhibitors (e.g., GANT61) demonstrate effectiveness in suppressing fibrosis and inflammation while restoring hormone sensitivity in preclinical studies, presenting highly promising targeted therapies	(Tam et al., 2022), (Jin et al., 2024), (El-Baz et al., 2022)
​	IL-24/IL-37 axle	IL-24 promotes epithelial-mesenchymal transition (EMT) via STAT3 and ERK1/2 pathways; IL-37 inhibits IL-24-induced EMT through the same signalling pathways	Modulates airway remodelling	Targeting IL-24 (e.g., with neutralising antibodies) or administration of exogenous IL-37 represents innovative strategies for reversing EMT and attenuating airway remodelling, though still at the experimental stage	Feng et al. (2022)
​	Rac1 signalling	Mediates airway smooth muscle proliferation and remodelling	Promotes airway remodelling	Rac1 inhibitors (e.g., NSC23766) effectively decrease remodelling in animal models with minimal adverse effects, representing promising therapeutic options for severe asthma	Dilasser et al. (2021)
Protease	Cathpsin C	Activates EMTU via the p38 pathway, contributing to airway remodelling	Promotes airway remodelling	The Cathpsin C inhibitor AZD7986, shown to prevent remodelling and inflammation, is currently in Phase III clinical trials for non-cystic fibrosis bronchiectasis	Yuan et al. (2024)
​	Cathpsin K	Stimulates EMTU through activation of the PAR2-mediated pathway	Promotes airway remodelling	Odanacatib, a Cathepsin K inhibitor, reduces EMTU activation and exhibits efficacy in animal asthma models. However, its development for osteoporosis was discontinued due to cardiovascular and cerebrovascular adverse effects, highlighting the necessity for safety considerations in future development	Qin et al. (2024)
Inflammasome	NLRP3 inflammasome	Exosomes derived from bone marrow-derived mesenchymal stem cells suppress inflammation and remodelling by targeting the NLRP3/ASC/Caspase-1/GSDMD pathway through miR-223-3p	Inhibits inflammation and airway remodelling	NLRP3 inflammasome inhibitors and stem cell-derived exosome therapies represent innovative approaches, though current research primarily remains in the preclinical phase	Li and Yang (2023)

### 4.1 Inflammatory response

PNECs regulate inflammatory responses through the release of neuropeptides and neurotransmitters following allergen exposure. Neuropeptides (substance P [SP], neurokinin A [NKA], vasoactive intestinal peptide [VIP], CGRP, and nociceptin/orphanin FQ [N/OFQ]) and neurotransmitters ((5-HT and GABA) exhibit diverse roles: some are pro-inflammatory, others anti-inflammatory, and certain mediators may bidirectionally modulate inflammation (Pavón-Romero et al., 2021).

#### 4.1.1 Neuropeptides

Substance P (SP) and neurokinin A (NKA) play central roles in airway inflammatory responses, primarily mediated through neurokinin-1 (NK-1R) and neurokinin-2 receptors (NK-2R) (Joos et al., 2000). SP exerts pro-inflammatory effects by binding to NK-1R on immune and epithelial cells. Asthma patients show heightened responsiveness to SP, with increased bronchial NK-1R expression (O'Connor et al., 2004; Li et al., 2022). NKA primarily mediates pro-inflammatory actions in asthma (Groneberg et al., 2004), although it can exhibit anti-inflammatory effects under specific conditions (Sumpter et al., 2014). Vasoactive intestinal peptide (VIP) is abundant in healthy lung of tissue (Onoue et al., 2007), modulating immune responses and exerting anti-inflammatory effects by interacting with specific receptors on immune cells (Misaka et al., 2010); thus, VIP functions as a potent anti-inflammatory mediator in asthma (Verma et al., 2017). Nociceptin/orphanin FQ (N/OFQ) may also demonstrate anti-inflammatory actions in severe asthma (D’Agostino et al., 2019).

CGRP can exert both pro-inflammatory and anti-inflammatory effects. The CGRP receptor consists of two components: a G protein-coupled receptor and a calcitonin gene-related peptide receptor-like (CLR) protein associated with receptor activity-modifying protein 1 (RAMP1). Studies indicate that the absence of the both receptors reduces inflammatory responses (Li et al., 2014). CGRP receptor antagonists suppress inflammation in allergic asthma by downregulating mouse ILC2s, supporting a pro-inflammatory role for CGRP (Ike et al., 2023). Conversely, in models such as acute lung injury, both endogenous and exogenous CGRP markedly attenuate inflammation, acting as key negative regulators of ILC2s activity to exert anti-inflammatory effects (Zhang R. et al., 2025). Clinically, the use of CGRP antagonists occasionally leads to inflammatory complications, suggesting that CGRP’s anti-inflammatory actions possess significant immunoregulatory value *in vivo*. These findings also highlight the complexity of CGRP-mediated immunomodulation. CGRP’s dual roles depend strongly on the tissue microenvironment, cell type, and signalling context, with its effects varying across experimental models and disease settings. Future studies must further elucidate its regulatory mechanisms and clinical implications.

#### 4.1.2 Neurotransmitters

The role of 5-HT in asthma is complex, as it can promote inflammation via certain mechanisms while exerting anti-inflammatory effects through activation of receptors such as 5-HT2A (Flanagan et al., 2017; Nau et al., 2012; Flanagan et al., 2024).

GABA also displays duality in asthma, possessing both anti-inflammatory and pro-inflammatory properties depending on receptor subtype, cell type, inflammatory milieu, and specific regulatory pathways. GABA and its receptors, particularly GABA_A receptors, are expressed on multiple immune cells (e.g., T cells, macrophages). Upon activation, they inhibit the production of inflammatory cytokines such as IL-6 and TNF-α, thereby reducing airway inflammation. Studies show that electroacupuncture-activated GABA signalling significantly attenuates airway inflammation in asthmatic mice by inhibiting the TLR4/MyD88/NF-κB pathway, an effect reversed by GABA_A receptor antagonists (Gong et al., 2023). Knockout of the GABA_A receptor α4 subunit enhances pulmonary inflammation and airway hyperreactivity in mouse asthma models, demonstrating the anti-inflammatory role of GABAergic signalling. Orally administered GABA_A receptor agonists (e.g., MIDD0301) effectively alleviate airway hyperresponsiveness and inflammation in asthmatic mice without inducing significant immunosuppression (Zahn et al., 2019). Notably, different GABA_A receptor subtypes exhibit distinct functional profiles. Activation of subtypes such as α1, α4, and α5 suppresses inflammation, whereas α3 activation may correlate with pro-inflammatory responses (Yocum et al., 2017). Although GABA signalling has limited direct impact on mucus secretion, it amplifies pathological mucus overproduction in the presence of inflammatory mediators like IL-13. Under such conditions, GABA_A receptor antagonists may be used to reduce mucus secretion. GABA_B receptors, as G protein-coupled receptors, regulate cAMP signalling and may influence epithelial and immune cell behaviour through paracrine mechanisms. In inflammatory conditions such as viral pneumonia, GABA_B receptor antagonists can attenuate inflammation, whereas agonists may worsen asthma symptoms, underscoring the need for precise therapeutic selection (Shirey et al., 2023). Future clinical applications of GABA-related therapies should be tailored to patient phenotypes and underlying pathological characteristics.

### 4.2 Airway remodeling

Airway remodeling represents another major pathological feature of asthma, holding significance equal to inflammation in the disease’s aetiology. Structural alterations in asthma include epithelial disruption, subepithelial fibrosis, goblet cell hyperplasia or metaplasia, smooth muscle hypertrophy or hyperplasia, and increased vascularization (Banno et al., 2020).

#### 4.2.1 Notch signalling

Studies demonstrate that following naphthalene-induced airway injury, rapid proliferation and hypertrophy of PNECs and NEBs occur during epithelial repair (Peake et al., 2000), indicating a significant role for PNECs in airway remodeling. Under conditions of airway injury, PNECs undergo transdifferentiation via activation of the Notch signaling pathway, thereby contributing to the regeneration of airway epithelial cells (Popko et al., 2020; Zhang et al., 2022). Notch signalling is a critical regulator of Ascl1, a transcription factor essential for PNEC formation and maintenance. Its inactivation can induce PNEC apoptosis through downregulation of Bcl2. Kim et al. demonstrated in mouse models that Notch signalling negatively regulates Ascl1 expression. Specifically, downregulation of Notch promotes PNEC proliferation, whereas Notch agonists suppress excessive proliferation, reducing airway reactivity and remodelling (Kim et al., 2025). Consequently, Notch agonists represent a promising therapeutic strategy to limit excessive PNEC proliferation and mitigate remodelling when combined with conventional asthma treatments.

#### 4.2.2 Epithelial-mesenchymal transition (EMT)

EMT constitutes a key mechanism underlying airway remodelling in chronic asthma. IL-24 promotes EMT in human bronchial epithelial cells (BEAS-2B) through activation of STAT3-and ERK1/2-dependent pathways, whereas IL-37 reverses this process, highlighting the IL-24/IL-37 axis as a potential therapeutic target to inhibit EMT and reduce airway remodelling. IL-37 exerts potent anti-inflammatory effects on innate and adaptive immunity. In a house dust mite (HDM)-induced mouse asthma model, IL-37 reduced airway eosinophil infiltration and remodelling by inhibiting IL-24-mediated EMT via modulation of STAT3 and ERK1/2 signalling. These findings identify IL-24 as a novel therapeutic target and IL-37 as a promising therapeutic agent for severe asthma (Feng et al., 2022).

#### 4.2.3 Rac1 signalling

Dilasser et al. demonstrated the potential role of Rac1 signalling in airway remodelling and airway smooth muscle cell (aSMC) proliferation in severe asthma, using human bronchial biopsies, cultured human aSMCs, and a severe allergic asthma mouse model sensitised to HDM. Pharmacological inhibition of Rac1 *in vivo* prevented airway remodelling associated with asthma, confirming Rac1 as a promising therapeutic target for severe asthma. Inhibiting Rac1-dependent signalling pathways concurrently reduced aSMC proliferation, airway hyperresponsiveness (AHR), and inflammation, presenting a novel approach to reverse airway remodelling and restore airway function in severe asthma patients. NSC23766, a Rac1 inhibitor, effectively mitigates airway remodelling with greater efficacy than some conventional therapies and minimal adverse effects (Dilasser et al., 2021).

#### 4.2.4 Cathepsin C/K

In severe asthma (SA) and uncontrolled asthma (UA), increased secretion of Cathepsin C by airway epithelial cells activates the epithelial-mesenchymal transition unit (EMTU) via p38-mediated pathways, thereby promoting airway remodelling. Yuan et al. screened differentially expressed genes in epithelial cells from SA and UA patients using RNA sequencing and identified Cathepsin C. Validation confirmed increased Cathepsin C levels in induced sputum samples from asthma patients, positively correlating with disease severity and airway remodelling. Direct instillation of exogenous Cathepsin C induced airway remodelling, whereas genetic suppression of Cathepsin C inhibited EMTU activation and remodelling. Therefore, Cathepsin C represents a promising biomarker and therapeutic target for SA or UA. AZD7986, a potent Cathepsin C inhibitor, prevents airway remodelling and inflammation in HDM-induced asthma mouse models. Currently in Phase III clinical trials for non-cystic fibrosis bronchiectasis (NCFBE), AZD7986 demonstrated manageable dental and cutaneous side effects at certain doses, not impeding clinical advancement (Yuan et al., 2024).

Elevated airway epithelial secretion of Cathepsin K activates EMTU through PAR2-mediated pathways. Qin et al. detected increased Cathepsin K levels in induced sputum from asthma patients (mild-to-moderate asthma [MMA] and SA), identifying it as a potential biomarker for airway remodelling. Experimental studies demonstrated significantly increased Cathepsin K expression in airway epithelium correlating with airway remodelling severity in HDM-induced mouse asthma models. Targeted inhibition of Cathepsin K suppressed EMTU activation and alleviated airway remodelling. Odanacatib, a specific CTSK inhibitor, effectively inhibited EMTU activation both *in vitro* and *in vivo* (Qin et al., 2024). Although odanacatib shows promise in asthma animal models, clinical data in asthma remain unavailable. Given its known cardiovascular and cerebrovascular side effects, future development of asthma treatments targeting Cathepsin K must prioritise target specificity and systemic safety.

#### 4.2.5 NLRP3 inflammasome

MiR-223-3p in mesenchymal stem cell-derived exosomes inhibits inflammation and remodelling via the NLRP3/ASC/Caspase-1/GSDMD pathway (Li and Yang, 2023). Stem cell-derived exosomes exhibit potent and targeted suppression of inflammation and remodelling. However, most studies remain limited to *in vitro* experiments and mouse models, with no available human clinical trial data. Moreover, optimal delivery routes (e.g., nebulisation, intravenous administration) and dosing strategies have yet to be systematically evaluated.

### 4.3 Airway hyperresponsiveness

Studies have shown that early respiratory exposure to arsenic exacerbates airway hyperresponsiveness in OVA-induced allergic asthma. Arsenic exposure further elevates 5-HT synthase levels and 5-HT content in the lungs of OVA-induced asthmatic mice. Upregulation of tryptophan hydroxylase (TPH)1 and TPH2,the two key 5-HT synthases, is markedly intensified in arsenic-exposed OVA-induced mice. LX1032, a specific TPH inhibitor, prevents the arsenic-induced exacerbation of airway hyperresponsiveness. These findings indicate that inhaled arsenic worsens airway hyperresponsiveness in OVA-induced allergic asthma by upregulating PNECs-derived 5-HT (Lu et al., 2025).

## 5 Therapeutic targets

### 5.1 Targeting PNEC secretions

**FIGURE 2:**
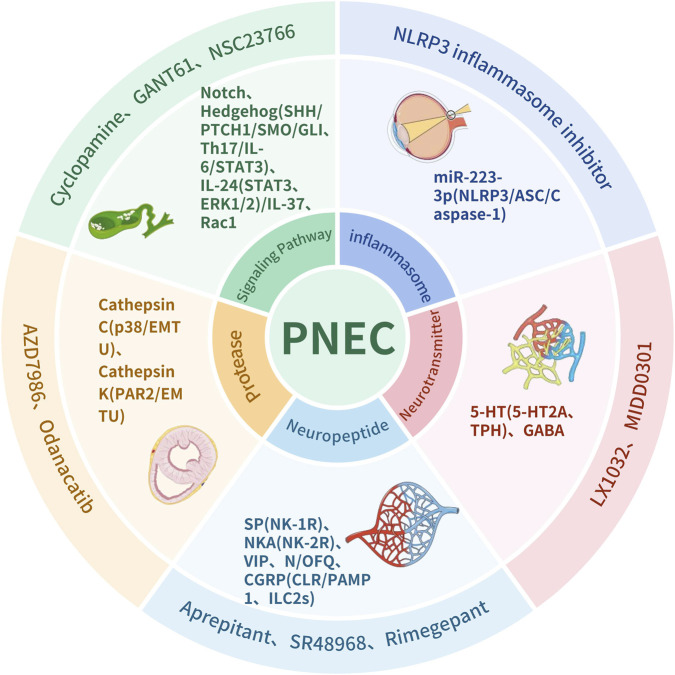
Schematic diagram of the PNEC-immune-neural network and its potential therapeutic targets. Abbreviations: SP, Substance P; NK-1R, Neurokinin-1 receptors; NKA, Neurokinin A; Neurokinin-2 receptors; VIP, Vasoactive intestinal peptide; N/OFQ Nociceptin/orphanin FQCGRP, Calcitonin gene-related peptide; CLR/PAMP1, Calcitonin Receptor-Like Receptor/Receptor Activity-Modifying Protein 1: Aprepitant, NK-1R antagonists: SR 48968, NK-2R antagonists; Aimegepant, CGRP receptor antagonists; 5-HT, Serotonin:5-HTZA, Serotonin Receptor 2A: TPH, Tryptophan hydroxylase; GABA,Gamma-aminobutyric acid: LX1032, TPH inhibitors: MIDD0301,GABA A receptor agonists miR-223-3P,microRNA-223-3p: NLRP3/ASC/Caspase-1, NOD-LRR-and Pyrin Domain-Containing Protein 3/Apoptosis-Associated Speck-Like Protein Containing a CARD/Cysteine-dependent Aspartate-Specific Protease-1; Notch, Neurogenic locus notch homolog protein; SHH, Sonic Hedgehog; PTCH1/SMO/GLI, Patched-1/Smoothened/Glioma-Associated Oncogene Homolog: STAT3, Signal Transducer and Activator of Transcription 3; ERK1/2, Extracellular Signal-Regulated Kinase 1/2; Rac1, Ras-related C3 botulinum toxin substrate 1: Cyclopamine, SMO inhibitors; GANT61, GLI inhibitors; NSC23766, Rac1 inhibitors:p38,p38 Mitogen-Activated Protein Kinase; EMTU, Epithelial-Mesenchymal Trophic Unit: PAR2, Protease-Activated Receptor 2; AZD7986, Cathpsin C inhibitor: Odanacatib, Cathepsin K inhibitor.

Increased PNEC numbers and enlarged NEB aggregates in asthma patients, accompanied by heightened responses to allergens, suggest that PNECs represent a viable therapeutic target. Approaches aimed at inhibiting PNEC secretions such as CGRP and GABA may offer new treatment options. Studies show that HDM exposure induces CGRP release from PNECs, which can be blocked by protease-activated receptor 1 (PAR1) antagonists, highlighting CGRP as a potential therapeutic target (Mann‐Nüttel et al., 2024). MIDD0301, a novel oral asthma therapy, reduces airway smooth muscle contraction and inflammation by targeting pulmonary GABA receptors without inducing systemic immunosuppression (Forkuo et al., 2018; Zahn et al., 2019). Futhan, a rapidly metabolised serine protease inhibitor, has been shown to reversibly inhibit the GABA_A receptors in A549 human alveolar epithelial cells (Chen et al., 2011) (Figure 2).

### 5.2 Interactions targeting PNECs with other cells

Targeting PNEC interactions with immune and epithelial cells, including the PNEC–ILC2 axis, PNEC–epithelial axis, and broader neuro-immune regulatory pathways, represents an emerging direction for novel asthma therapies, with several compounds demonstrating promising effects in animal models.

PNECs secrete CGRP to directly activate ILC2s, promoting the release of inflammatory mediators such as IL-5 and driving airway inflammation. Rimegepant, a CGRP receptor antagonist, effectively alleviates allergic asthma symptoms in mice by inhibiting the PNEC–CGRP–ILC2s pathway (Ike et al., 2023). PNEC-secreted GABA induces goblet cell hyperplasia and excessive mucus secretion—a key mechanism underlying airway remodelling and symptom exacerbation in asthma. GABA signalling inhibitors can block the PNEC–GABA–goblet cell axis by antagonising GABA_A or GABA_B receptors, thereby reducing mucus production (Thakur et al., 2024). PNECs also interact with neurons via the vagus nerve and TRPA1 pathways to regulate airway reactivity and inflammation. Targeting molecules such as TRPA1 and NT4 can disrupt neurogenic regulation of PNEC secretion, reducing excessive mucus production. Bronchial thermoplasty attenuates airway inflammation by disrupting the vagal ganglion TRPA1–PNEC axis through NRG1–ERBB signalling between PNECs and neighbouring cells (Feng et al., 2025).

## 6 Discussion

Asthma is a complex chronic inflammatory airway disease involving intricate interactions among various cells and molecules. PNECs have attracted increasing attention in asthma research due to their unique biological characteristics and extensive intercellular interactions. Current studies suggest that targeting PNECs could offer novel therapeutic approaches for asthma management. However, the precise regulatory mechanisms and their differential contributions to distinct asthma phenotypes require further investigation. Existing research primarily focuses on eosinophilic (allergic) asthma, whereas the specific roles and regulatory mechanisms of PNECs in neutrophilic (non-type 2) asthma remain underexplored. PNECs are implicated in various other diseases, including COPD and lung cancer. Targeting PNECs may induce non-specific adverse effects, necessitating vigilance regarding potential risks. PNECs participate not only in inflammatory responses but also in airway injury repair and regeneration. The functions of PNECs in airway epithelial regeneration, fibrosis, and interactions with stem and stromal cells remain largely unexplored. Multiple molecules secreted by PNECs exhibit overlapping functions; intervention at a single target may be compensated by alternative pathways, thereby compromising therapeutic efficacy. Additionally, the lack of effective functional testing systems for human-derived PNECs and large-scale clinical data has hindered the translation of fundamental discoveries into clinical applications. Interventions targeting PNECs have yet to advance into clinical evaluation, indicating a substantial translational gap.

This gap may be attributed to several factors. Firstly, the heterogeneity of PNECs across different airway compartments remains inadequately characterised, complicating target identification and the elucidation of underlying mechanisms. Secondly, the absence of reliable biomarkers impedes patient stratification and efficacy monitoring, as demonstrated by the lack of standardised detection methods for CGRP and GABA in exhaled breath condensate (EBC). Thirdly, the complex interactions through which environmental exposures (e.g., pollutants, cigarette smoke, and e-cigarettes) influence PNEC activation and downstream immune responses, coupled with significant population heterogeneity, further increase uncertainty in clinical translation. Moreover, PNECs constitute an extremely small fraction of the pulmonary epithelium, presenting significant challenges in isolation, purification, and *in vitro* culture. This limitation has restricted research involving human samples, with findings predominantly relying on animal models, thereby carrying inherent risks of species-specific differences. Existing studies are largely based on mouse or primate models; certain gene knockout or overexpression models may inadequately replicate human asthma’s complexity, necessitating cautious extrapolation. Current animal models and traditional 2D cell cultures inadequately replicate the complexity of the human PNEC–immune axis, lacking high-fidelity *in vitro* systems suitable for mechanistic exploration and drug screening.

To accelerate the clinical translation of therapies targeting the PNEC–immune axis, future research should employ single-cell sequencing and spatial omics technologies to systematically characterise the molecular features, distribution, and interactions of PNECs with immune cells across different airway regions. Single-cell sequencing has revealed multiple PNEC subtypes with distinct gene expression and functional characteristics under disease conditions. Future studies should clarify the specific roles of each subtype within different asthma phenotypes and their interactions with immune cells. Existing research indicates that PNECs amplify airway inflammatory responses by regulating ILC2s and goblet cells via neuropeptides (e.g., CGRP) and neurotransmitters (e.g., GABA). Future investigations should explore the bidirectional regulatory networks between PNECs and the nervous and immune systems, particularly focusing on interactions with the vagus nerve and Trpv1+ neurons, and their effects on airway hyperresponsiveness. Given the significantly elevated numbers and activity of PNECs in asthma patients, targeting molecules such as CGRP and GABA secreted by PNECs could serve as promising novel therapies. Future research should evaluate pharmacological interventions targeting PNEC-related signalling pathways and their efficacy across different asthma phenotypes. Detection methods for biomarkers (e.g., CGRP and GABA in EBC) require further standardisation, complemented by multi-omics approaches to identify novel PNEC-associated biomarkers for non-invasive diagnosis and treatment prediction. Systematic investigations are also necessary to clarify how environmental factors, including pollutants, cigarette smoke, and e-cigarettes, affect PNEC activation, secretion, and immune modulation, elucidating their roles in asthma susceptibility and progression. Additionally, organoid and 3D co-culture systems should be developed to reconstruct the PNEC–immune microenvironment. Combined with PNEC-specific inhibitors, these advanced models will facilitate in-depth mechanistic studies and robust drug screening, thereby enhancing translational potential. Advances in human-derived PNEC culture and organoid technologies now allow better simulation of human disease environments, thereby accelerating the translation of basic research into clinical applications.

In summary, clinical translation of strategies targeting the PNEC–immune axis urgently requires integrated advancement in fundamental and applied research. PNECs, as pivotal cells in asthma pathogenesis, hold significant research value due to their extensive intercellular interactions and unique neuroendocrine-immune functions. Future research should focus on their functional subtypes, neuro-immune regulatory networks, targeted therapies, and clinical translation. Concurrently, vigilance is required regarding model limitations, detection challenges, and potential adverse effects. While actively pursuing breakthroughs, technical limitations and clinical risks must be cautiously addressed to advance this field towards greater precision and personalisation. Progress in areas such as heterogeneity analysis, biomarker development, elucidation of environmental mechanisms, and high-fidelity *in vitro* modelling will establish a robust foundation for developing innovative therapies targeting the PNEC–immune axis.

## Glossary

PNECs: Pulmonary neuroendocrine cells
ILC2s: type 2 innate lymphoid cells
GC: Goblet cells
Eos: Eosinophils
ICS: Inhaled corticosteroids
CGRP: Calcitonin gene-related peptide
GABA: Gamma-aminobutyric acid
5-HT: Serotonin/5-hydroxytryptamine
SP: Substance P
NKA: Neurokinin A
N/OFQ: Nociceptin/orphanin FQ
VIP: Vasoactive intestinal peptide
NEBs: Neuroepithelial bodies
HPV: Hypoxic pulmonary vasoconstriction
DCVs: Dense cisternae vesicles
ACh: Acetylcholine
Ascl1: Achaete-scute-like protein 1
IL: Interleukin
SHH: Sonic Hedgehog
PAH: Pulmonary hypertension
MUC: Mucin
MBP: Major basic protein
EETs: Eosinophil extracellular traps
TSLP: Thymic stromal lymphopoietin
NK-1R: Neurokinin-1 receptors
NK-2R: Neurokinin-2 receptors
TPH: Tryptophan hydroxylase
AI: Airway Inflammation
AR: Airway Remodelling
AHR: Airway Hyper-responsiveness
MEC: Myoepithelial Cell
ECs: Epithelial Cells
